# The Impact of Early Life Experiences and Gut Microbiota on Neurobehavioral Development in Preterm Infants: A Longitudinal Cohort Study

**DOI:** 10.3390/microorganisms11030814

**Published:** 2023-03-22

**Authors:** Jie Chen, Hongfei Li, Tingting Zhao, Kun Chen, Ming-Hui Chen, Zhe Sun, Wanli Xu, Kendra Maas, Barry M. Lester, Xiaomei S. Cong

**Affiliations:** 1 College of Nursing, Florida State University, Tallahassee, FL 32306, USA; 2 School of Nursing, University of Connecticut, Storrs, CT 06269, USA; 3 Department of Statistics, University of Connecticut, Storrs, CT 06269, USA; 4 School of Nursing, Yale University, Orange, CT 06477, USA; 5 Department of Biostatistics, School of Public Health, Yale University, New Haven, CT 06520, USA; 6 Microbial Analysis, Resources, and Services (MARS), University of Connecticut, Storrs, CT 06269, USA; 7 Brown Center for the Study of Children at Risk, Departments of Psychiatry and Pediatrics, Warren Alpert Medical School of Brown University, Providence, RI 02903, USA; 8 Institute for Systems Genomics, University of Connecticut, Farmington, CT 06030, USA

**Keywords:** infants, preterm, NICU, neurobehavioral development, gut microbiota, pain, stress, feeding

## Abstract

Objectives: The objective of this study is to investigate the impact of early life experiences and gut microbiota on neurobehavioral development in preterm infants during neonatal intensive care unit (NICU) hospitalization. Methods: Preterm infants were followed from NICU admission until their 28th postnatal day or until discharge. Daily stool samples, painful/stressful experiences, feeding patterns, and other clinical and demographic data were collected. Gut microbiota was profiled using 16S rRNA sequencing, and operational taxonomic units (OTUs) were selected to predict the neurobehaviors. The neurobehavioral development was assessed by the Neonatal Neurobehavioral Scale (NNNS) at 36 to 38 weeks of post-menstrual age (PMA). Fifty-five infants who had NNNS measurements were included in the sparse log-contrast regression analysis. Results: Preterm infants who experienced a high level of pain/stress during the NICU hospitalization had higher NNNS stress/abstinence scores. Eight operational taxonomic units (OTUs) were identified to be associated with NNNS subscales after controlling demographic and clinical features, feeding patterns, and painful/stressful experiences. These OTUs and taxa belonging to seven genera, i.e., *Enterobacteriaceae_unclassified*, *Escherichia-Shigella*, *Incertae_Sedis*, *Veillonella*, *Enterococcus*, *Clostridium_sensu_stricto_1*, and *Streptococcus* with five belonging to *Firmicutes* and two belonging to *Proteobacteria* phylum. The enriched abundance of *Enterobacteriaceae_unclassified* (OTU17) and *Streptococcus* (OTU28) were consistently associated with less optimal neurobehavioral outcomes. The other six OTUs were also associated with infant neurobehavioral responses depending on days at NICU stay. Conclusions: This study explored the dynamic impact of specific OTUs on neurobehavioral development in preterm infants after controlling for early life experiences, i.e., acute and chronic pain/stress and feeding in the NICU. The gut microbiota and acute pain/stressful experiences dynamically impact the neurobehavioral development in preterm infants during their NICU hospitalization.

## 1. Introduction

The mortality rate of preterm infants has significantly decreased in recent years alongside the advances of neonatal healthcare and medical treatments [1,2,3], whereas preterm infants are still at a high risk of neurodevelopmental deficiency in early life as well as late childhood mortality and late-onset mental and behavioral disorders [4,5,6]. The prevention of neurodevelopmental deficiencies in these infants has been placed at the forefront of child healthcare issues [7]. Current interventional strategies in promoting neurodevelopment in preterm infants are still lacking and less than optimal because the underlying mechanisms of neurobehavioral development are understudied in these high-risk populations, which hinders the timely prevention, treatment, and prediction of neurobehavioral deficiencies in the early life stages.

The etiologies of preterm infant neurodevelopment are complex and multifactorial. We recently found that cumulative pain/stress experiences in early life are significantly related to altered neurobehavioral responses in preterm infants [4], but the mechanisms demand further investigation. The brain–gut–microbiome axis, in which the intestinal microbiome is proposed to play a key role in the regulation of stress and early programming of the neuro-immune system that has been found to influence all aspects of human behaviors [8,9,10]. Preclinical and clinical studies have shown the brain–gut–microbiome axis involved in the regulation of neurobehavioral and cognitive development [10,11]. Studies have reported that the gut microbiota regulates the pathophysiologic process of brain injury and neurological developments in preterm infants [9,12,13]. Several gut bacteria species have been identified as being involved in behavior mitigation and cognitive adjustment [14,15].

Identifying potential pathogens and the pathogenesis process of gut microbiota involved in neurobehavioral development in preterm infants will facilitate the early relief and treatment of neurobehavioral deficiencies. Much is still unknown regarding the impact of early life experiences combined with gut microbiota on neurobehavioral development in preterm infants and few studies have used a longitudinal cohort design. Therefore, our study aimed to explore the longitudinal impact of gut microbiota and daily painful/stress experiences on the neurobehavioral development in preterm infants during NICU hospitalization.

## 2. Materials and Methods

### 2.1. Design

A longitudinal cohort study was conducted at two NICUs in the northeastern U.S. from January 2014 to August 2017. Preterm infants were followed from admission into the NICUs until their 28th postnatal day or discharge from the NICUs. The study protocol was approved by the institutional review board of the study hospital and the affiliated institute. Written informed consent was obtained from parents of the preterm infants.

### 2.2. Inclusion and Exclusion Criteria

Preterm infants were included if they were: (1) 0–7 days old after birth, (2) born at 28 to 32 weeks of gestational age (28 0/7 to 32 6/7), and (3) had a negative drug exposure history (no illicit drug use during pregnancy). Exclusion criteria included: (1) infant mothers that were younger than 18 years old, (2) severe periventricular/intraventricular hemorrhage (≥Grade III), and (3) other known congenital anomalies.

### 2.3. Measurements

#### 2.3.1. Demographic and Clinical Data Collection

Demographic and clinical characteristics including sex, gestational age (GA), delivery type, and birth weight and length were recorded by research nurses. The severity of illness of the infant was measured using the Score for Neonatal Acute Physiology—Perinatal Extension-II (SNAPPE-II) [16]. Daily antibiotic use, feeding types (mother’s breast milk, donor’s milk, and formula milk) and frequency and painful/stress experiences during NICU hospitalization were recorded by research nurses.

#### 2.3.2. Assessment of Daily Painful/Stressful Experiences

The Neonatal Infant Stressor Scale (NISS) was used to assess daily painful/stress experiences in early life, which was modified from the Australian version in our previous study based on the NICU practice in the U.S. [4]. The NISS includes 47 acute events (e.g., diaper change, X-ray, intravenous (IV) injection, etc.) and 23 chronic procedures (e.g., intranasal oxygen, nil per os, etc.). The intensities of the acute and chronic painful/stressful procedures are categorized into five domains (1 = not painful/stressful, 2 = a little painful/stressful, 3 = moderately painful/stressful, 4 = very painful/stressful, and 5 = extremely painful/stressful), such as the extremely painful/stressful procedures and events (level 5) including multiple intravenous infusion (acute), and a little painful/stressful procedures and events (level 2) including mouth care (acute) and high-flow nasal cannula oxygen (chronic). The detailed painful/stressful procedures and pain severity levels are listed in Supplementary File S1.

Trained research nurses extracted the frequencies of the defined acute painful/stressful events and hours of the defined chronic painful/stressful procedures from the infant electronic medical record and documented these data into the Research Electronic Data Capture (REDCap) system [17] to generate daily NISS data for each infant. The data were audited following our protocol. Weighted frequencies (acute) and hours of procedures (chronic) were calculated by timing the counts and intensities of each procedure in each day of NICU stay to generate daily acute pain/stress scores and chronic pain/stress scores following our protocol [4].

#### 2.3.3. Fecal Sample and Gut Microbiota

The fecal sample collection, DNA extraction, and processing followed our previous methods and procedures [18,19]. Daily fecal samples were collected during diaper change depending on whether an infant had stool. Fecal samples were placed into a sterile specimen container (5 mL) and transferred into a −80 freezer immediately. Then, 0.25 g fecal samples were aliquoted into bead tubes for DNA extraction using the MoBio Power Soil kit (MoBio Laboratories, Inc., Carlsbad, CA, USA) according to the manufacturer’s instruction for the Eppendorf epMotion 5075 Vac liquid handling robot or manually. DNA extracts were quantified using a Synergy HT (Biotek, Winooski, VT, USA) with the Quant-iT PicoGreen kit (Invitrogen, ThermoFisher Scientific, Waltham, MA, USA). The V4 regions of the 16S rRNA gene were amplified with 515F and 806R primers containing Illumina adapters and golay indices on the 3′ end using 20 ng of extracted DNA as a template. Samples were amplified in triplicate using Phusion High-Fidelity PCR master mix (New England BioLabs, Ipswich, MA, USA) with the addition of 10 μg BSA (New England BioLabs). The PCR reaction was incubated at 95 °C for 3.5 min with 30 cycles of 30 s at 95.0 °C, 30 s at 50.0 °C, and 90 s at 72.0 °C, followed by a final extension at 72.0 °C for 10 min. PCR products were quantified and visualized using the QIAxcel DNA Fast Analysis (Qiagen, Hilden, Germany). PCR products were normalized based on the concentration of DNA in the 350–400 bp region and pooled using the QIAgility liquid handling robot (Qiagen). Pooled PCR products were cleaned using the Gene Read Size Selection kit (Qiagen) according to the manufacturer’s protocol. The cleaned pool was sequenced with the MiSeq using a v2 2 × 250 base-pair kit (Illumina, Inc., San Diego, CA, USA).

The raw sequence data were processed by the Mothur 1.42.3 pipeline [20] following the Mothur miseq process and the miseq bash (Supplementary File S2) [19]. This process began by assembling paired end reads into contigs. A quality score was set for removing low quality reads. The operational taxonomic units (OTUs) were determined by clustering reads to the SILVA 119 16S reference dataset at a 97% identity, and then performing de novo OTU clustering on reads that failed to cluster to a reference [21]. The chimeric sequences were also removed. Taxonomic annotation was also determined by the SILVA 119 V4 16 S rRNA reference database [22,23].

#### 2.3.4. Neurobehavioral Development Assessment

Neurobehavioral outcomes were assessed using the NICU Network Neurobehavioral Scale (NNNS) [24] when an infant reached 36 to 38 weeks post-menstrual age (PMA) before the NICU discharge. The NNNS includes 115 items resulting in 13 summary scores assessing habituation, attention, arousal, self-regulation, handling, quality of movement, excitability, lethargy, reflexes, asymmetrical responses, hypertonicity, hypotonicity, and stress/abstinence. One trained and certified NNNS examiner who was blinded to all other assessments completed all the assessment and scoring of the NNNS subscales.

### 2.4. Data Analysis

The demographic and clinical data and OTU tables were imported into R 4.0.0 for statistical analysis. The clinical variables including the painful/stressful procedures of different levels and the population daily feeding of mother’s breast milk, donor’s milk, and formula milk were visualized by plotting the pattern over time using the “ggplot2” package in R [25]. The sex differences regarding the demographic and clinical characteristics were tested by Wilcoxon rank sum test for continuous variables and Fisher’s exact test for categorical variables.

To explore the predictive microbiome biomarkers and estimate the time-varying dynamics of their impact during the early postnatal stage on the neurobehavioral outcomes of the preterm infants, sparse log-contrast regression with functional compositional predictors [26] was adopted. Infants who had five or more fecal samples after raw sequencing data processing were included in the current analysis to explore the time-varying effects using the sparse log-contrast regression model. The core OTUs were screened by the abundance and prevalence criteria before fitting the statistical model. Demographics variables including sex and race, delivery type, premature rupture of membranes (PROM) status, and gestational age at birth were incorporated into the model as time-invariant control variables. The cubic spline basis was used for modeling the time-varying effects of the OTUs and a constrained group lasso (CGL) algorithm was used for compositional component selection at OTU level [26,27]. One hundred bootstrap samples were generated and used to provide supporting evidence for the stability of the results. The OTUs were chosen by the model selection process and those with higher proportions of being selected in the bootstrap procedure were kept.

## 3. Results

### 3.1. Demographic and Clinical Characteristics

A total of 92 preterm infants were recruited, and 55 infants were included in this report based on the completion of the microbiome and NNNS measurements (Supplementary Figure S1). The majority of infants were non-Hispanic/Latino White (74.55%), female (54.55%), and born via C-section (61.82%) (Table 1). About 80% of the preterm infants received antibiotics during the first 3 days of the NICU stay; after day 3, only 20% of them used antibiotics. Feeding patterns included mother’s breast milk breastfeeding (61.65%), human donor milk (26.31%), and formula milk (12.04%) during the NICU hospitalization. The proportion of mother’s breast milk intake are shown in Supplementary Figure S2a, and sex-specific daily feeding patterns are shown in Supplementary Figure S3. For the daily average painful/stress experience (NISS scores), weighted frequencies of acute painful events (mean = 62.66, SD = 9.94) and weighted hours of chronic painful procedures (mean = 89.84, SD = 36.72) were calculated and plotted across sex (Supplementary Figure S2b,c).

### 3.2. The Gut Microbiota Compositions

**Figure 1:**
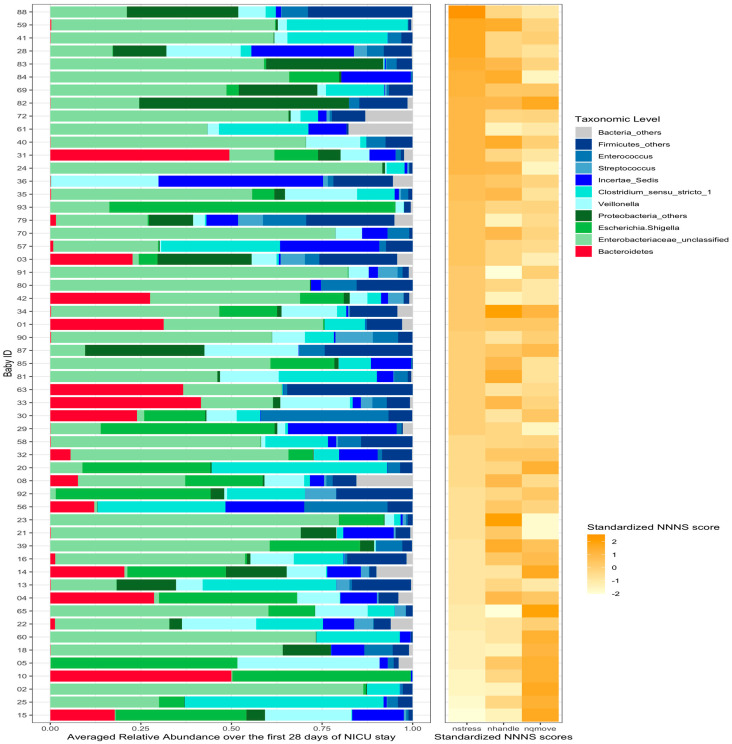
Relative abundance of gut microbiota and 3 sub-scales for each infant. The infants were ordered according to the standardized score of NSTRESS scores. A standardized score of NNNS subscales (NSTRESS, NQMOVE, and NHANDLING) was generated by dividing the difference between each infant’s score and the mean by the standard deviation. The standardized scores of each of these subscales were plotted with the gut microbiome for each infant.

A total of 584 stool samples were included in the analysis (Supplementary Table S1). The most abundant phyla were *Proteobacteria*, *Firmicutes*, and *Bacteroidetes*. The compositional relative abundances for the 55 preterm infants were plotted on an average basis (Figure 1). Detailed taxonomy of each OTU was summarized in Supplementary Table S2.

### 3.3. Neurobehavioral Development

The NNNS assessment scores were presented in Table 2 and Supplementary Figure S4. These preterm infants had high levels of hypertonicity, hypotonicity, and asymmetric reflexes (median score = 0) followed by stress/abstinence and handling (Supplementary Figure S4). Given the substantial amount of missing values in some of the subscales, the main focuses of the current analysis were on stress/abstinence (NSTRESS), handling (NHANDLING), and quality of movement (NQMOVE). The stress/abstinence (NSTRESS), handling (NHANDLING), and quality of movement (NQMOVE) in these preterm infants at 36 to 38 weeks of post-menstrual age were 0.18 (SD = 0.09), 0.56 (SD = 0.21), and 3.97 (SD = 0.62), respectively. There was no significant difference between females and males.

### 3.4. Associations of Pain/Stress Experience and Gut Microbiota with Neurobehaviors

**Figure 2:**
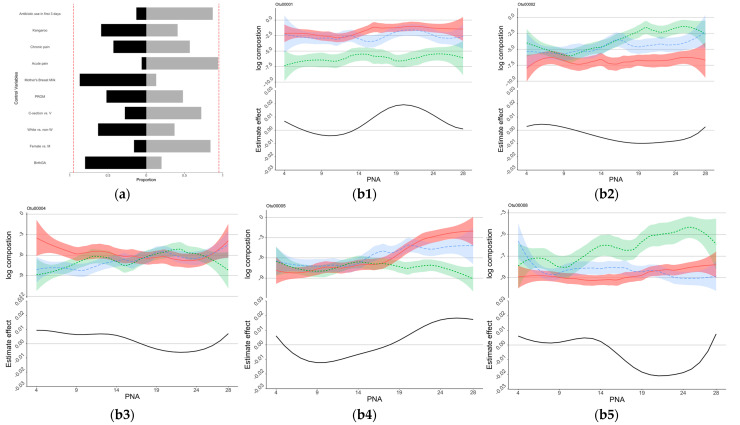

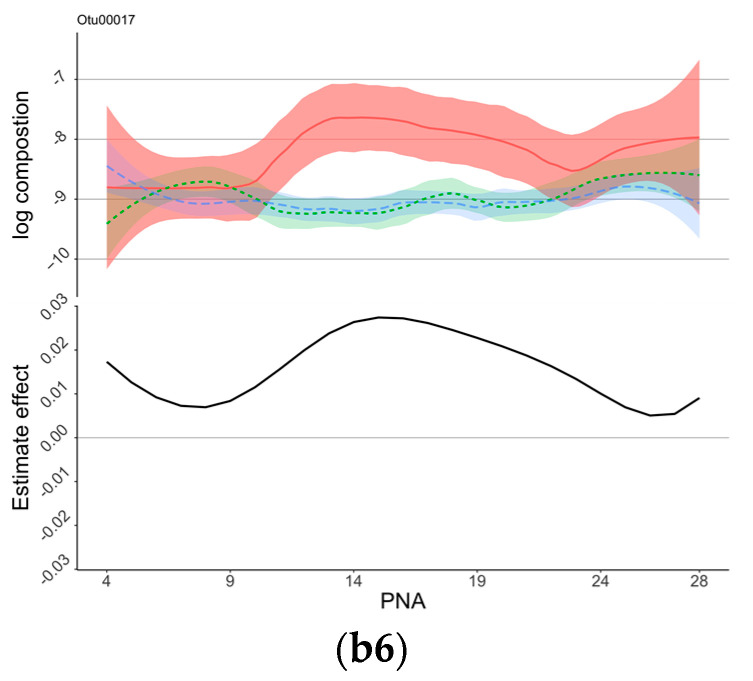
Control sign and OTUs for NSTRESS. (**a**) Control sign and NSTRESS. (**b**) OTUs associated with NSTRESS scores. (**a**) The proportions of the signs of the estimated coefficients of the control variables. Proportions of negative signs were shown as black blocks to the right, and those of positive signs were shown as light gray blocks to the left. Lower NSTRESS scores indicated better development, those variables in black indicated association with lower NSTRESS scores. The red dotted lines show the 90% of bootstrap. (**b1**–**b6**) The estimated time-varying effects of OTU on NSTRESS scores during the NICU stay. The *x*-axis represents the days after birth (PNA, postnatal age), the *y*-axis represents the log composition of the OTU abundance (top panel) and the estimated effect (bottom panel). The top panel “log composition” displays the trend of the log-transformed OTU compositions over the first 28 days of NICU stay for infants with different NSTRESS scores, with the infants being separated into three groups based on their NSTRESS score: high NSTRESS score (plotted in red), medium NSTRESS score (plotted in green), and low NSTRESS score (plotted in blue). The bottom panel “Estimate effect” is the estimated time-varying effects of OTUs on NSTRESS scores during the NICU stay.

**Figure 3:**
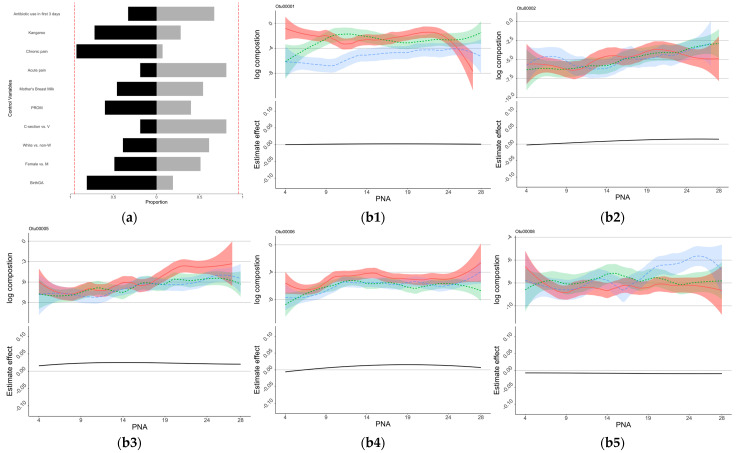

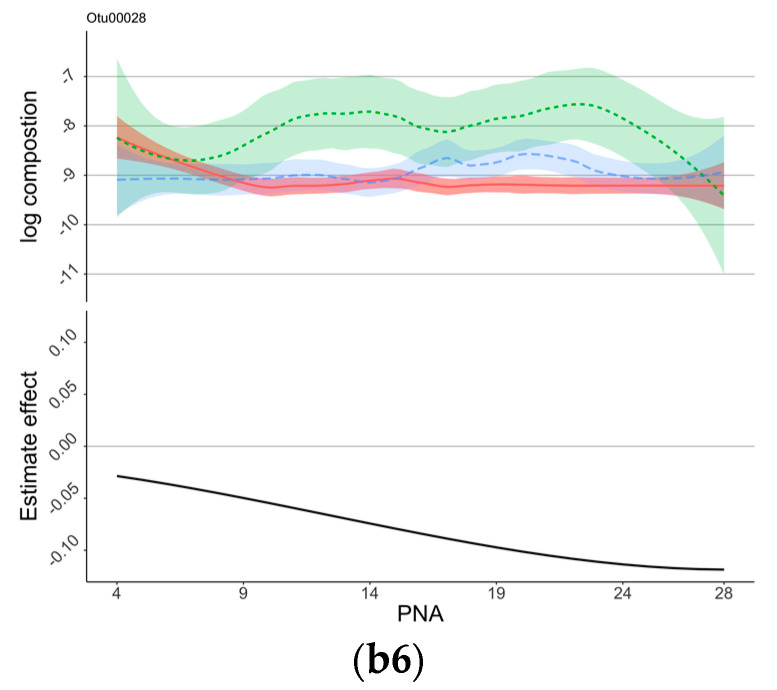
Control sign and OTUs for NHANDLING. (**a**) Control sign and NHANDLING. (**b**) OTUs associated with NHANDLING scores. (**a**) The proportions of the signs of the estimated coefficients of the control variables. Proportions of negative signs were shown as black blocks to the right, and those of positive signs were shown as light gray blocks to the left. Lower NHANDLING scores indicated better development, those variables in black indicated association with lower NHANDLING scores. The red dotted lines show the 90% of bootstrap. (**b1**–**b6**) The estimated time-varying effects of OTU on NSTRESS scores during the NICU stay. The *x*-axis represents the days after birth (PNA, postnatal age), the *y*-axis represents the log composition of the OTU abundance (top panel) and the estimated effect (bottom panel). The top panel “log composition” displays the trend of the log-transformed OTU compositions over the first 28 days of NICU stay for infants with different NHANDLING scores, with the infants being separated into three groups based on their NHANDLING score: High NHANDLING score (plotted in red), medium NHANDLING score (plotted in green), and low NHANDLING score (plotted in blue). The bottom panel “Estimate effect” is the estimated time-varying effects of OTUs on NHANDLING scores during the NICU stay.

**Figure 4:**
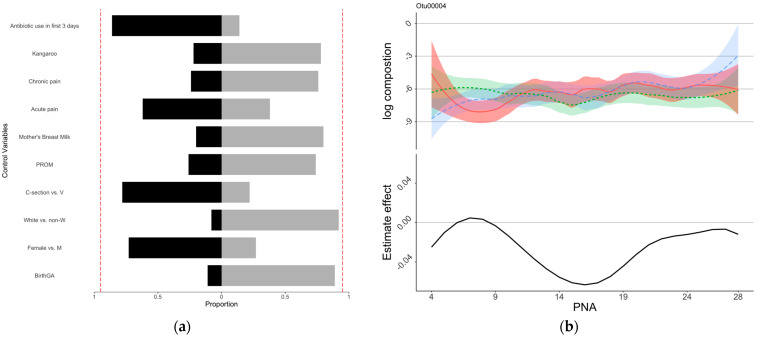
Control sign and OTUs for NQMOVE. (**a**) Control sign and NQMOVE. (**b**) OTUs associated with NQMOVE scores. (**a**) The proportions of the signs of the estimated coefficients of the control variables. Proportions of negative signs were shown as black blocks to the right, and those of positive signs were shown as light gray blocks to the left. Higher NQMOVE scores indicated better development, those variables in light gray indicated association with higher NQMOVE scores. The red dotted lines show the 90% of bootstrap. (**b**) The estimated time-varying effects of OTU on NQMOVE scores during the NICU stay. The *x*-axis represents the days after birth (PNA, postnatal age), the *y*-axis represents the log composition of the OTU abundance (top panel) and the estimated effect (bottom panel). The top panel “log composition” displays the trend of the log-transformed OTU compositions over the first 28 days of NICU stay for infants with different NQMOVE scores, with the infants being separated into three groups based on their NQMOVE score: high NQMOVE score (plotted in red), medium NQMOVE score (plotted in green), and low NQMOVE score (plotted in blue). The bottom panel “Estimate effect” is the estimated time-varying effects of OTU on NQMOVE scores during the NICU stay.

The estimated coefficients of the control variables for the NSTRESS, NQMOVE, and NHANDLING assessment are shown in Supplementary Table S3. As shown by the bootstrap analysis, infants with older birth GA, male sex, white race, vaginal birth, no PROM, lower acute pain, higher kangaroo care, and no antibiotic use in the first 3 days of NICU stay might be associated with better outcomes including lower NSTRESS scores (Figure 2a), lower NHANDLING scores (Figure 3a), and higher NQMOVE scores (Figure 4a). In particular, a positive association between higher acute pain/stress (NISS score) and higher NSTRESS scores was seen in close to 95% of the bootstrap (Figure 2a), indicating that infants who experienced less acute painful/stressful events during the NICU stay had better neurobehavioral outcomes. However, the relationships between feeding patterns and chronic pain (NISS score) and NNNS subscales are still undetermined.

To illustrate the relationships between gut microbiota compositions and NNNS subscales (NSTRESS, NHANDLING, and NQMOVE), the standardized scores of each these subscales were plotted with the gut microbiome for each infant using a heatmap (Figure 1). A standardized score of the NNNS subscales was generated by dividing the difference between each infant’s score and the mean by the standard deviation.

Eight OTUs were identified to be associated with NNNS subscales through the regression analysis (Figure 2, Figure 3 and Figure 4, and Supplementary Table S4). At the taxonomy levels, five belong to *Firmicutes* (OTU4, OTU5, OTU6, OTU8, and OTU28), and three belong to *Proteobacteria* (OTU1, OTU2, and OTU17). The taxa of OTU1 (*Enterobacteriaceae_unclassified*), OTU2 (*Escherichia-Shigella*), and OTU17 (*Enterobacteriaceae_unclassified*) were identical at the family level (*Enterobacteriaceae*); OTU1 (*Enterobacteriaceae_unclassified*) and OTU17 (*Enterobacteriaceae_unclassified*) were also identical at the genus level (Supplementary Table S2). The associations between these OTUs and the NNNS subscales varied depending on the day of NICU stay (Figure 2, Figure 3 and Figure 4) after controlling for feeding types and pain/stress experiences in addition to demographic and clinical characteristics.

OTU1 (*Enterobacteriaceae_unclassified*), OTU2 (*Escherichia-Shigella*), OTU5 (*Veillonella*), OTU4 (*Incertae_Sedis*), OTU8 (*Clostridium_sensu_stricto_1*), and OTU17 (*Enterobacteriaceae_unclassified*) were identified to be associated with NSTRESS, and their estimated time-varying effects are each presented in the subfigures of Figure 2. The effect of OTU2 (*Escherichia-Shigella*), OTU4 (*Incertae_Sedis*), and OTU8 (*Clostridium_sensu_stricto_1*) on the NSTRESS score switches from positive to negative during the postnatal days from 4 to 28, whereas the effects of OTU1 (*Enterobacteriaceae_unclassified*) and OTU5 (*Veillonella*) switch from negative to positive. OTU17 (*Enterobacteriaceae_unclassified*) shows consistently positive effect on NSTRESS score during the 28 days of NICU stay. Elevated abundance of *Enterobacteriaceae* (OTU1 and OTU17) was significantly associated with increased NSTRESS, particularly after two weeks of NICU stay (Figure 2). However, the elevated enrichment of *Escherichia-Shigella* (OTU2) was associated with decreased NSTRESS, particularly after 10 days.

OTU1 (*Enterobacteriaceae_unclassified*), OTU2 (*Escherichia-Shigella*), OTU 5 (*Veillonella*), OTU 6 (*Enterococcus*), OTU8 (*Clostridium_sensu_stricto_1*), and OTU28 (*Streptococcus*) were selected for the model regressing on NHANDLING. Their estimated time-varying effects are presented in each subfigures of Figure 3. The effect of OTU1 (*Enterobacteriaceae_unclassified*), OTU2 (*Escherichia-Shigella*), OTU5 (*Veillonella*), and OTU6 (*Enterococcus*) on the NHANDLING score remained positive during the first month, whereas the effect of OTU8 (*Clostridium_sensu_stricto_1*) was constantly negative. OTU28 (*Streptococcus*) shows an enlarging negative effect on the NHANDLING score over the first month.

OTU4 (*Incertae_Sedis*) was the only OTU selected for NQMOVE; its estimated time-varying effects are presented in Figure 4. The effect of OTU4 (*Incertae_Sedis*) on the NQMOVE score became negative after day 10.

## 4. Discussion

Our study demonstrated the impact of early life pain/stress experience and gut microbiota on neurobehavioral outcomes in preterm infants during their NICU hospitalization using a longitudinal modeling approach. Consistent with previous studies, our findings showed that preterm infants had a higher risk of neurobehavioral deficiency than full-term infants [28,29,30]. In comparison to the neurobehavioral results from healthy full-term infants at birth [28], our findings showed that preterm infants had higher NNNS scores than full-term infants in stress/abstinence (0.18 vs. 0.11) and handling responses (0.56 vs. 0.38) and lower quality of movement (3.97 vs. 4.71). The negative impact of higher acute painful/stressful events during the NICU stay on worse neurobehavioral outcomes is congruent with previous studies [3,31,32]. We identified eight OTUs of the gut microbiome that were significantly associated with infant neurobehavioral profiles in early life. Most importantly, our study uncovered potential pathogenesis process of *Enterobacteriaceae* and *Streptococcaceae* involved in neurobehavioral outcomes by depicting the dynamical impacts of OTUs on NNNS scores. These findings are consistent with previous studies, which showed that the brain–gut–microbiome axis is involved in neonatal brain damage and immunity [9,33] and influences the lifelong health of humans [34,35].

The role of Enterobacteriaceae on NSTRESS is still unclear given that elevated abundance of *Enterobacteriaceae_unclassified* (OTU1 and OTU17) was significantly associated with increased NSTRESS, but the elevated enrichment of Escherichia-Shigella (OTU2) was associated with decreased NSTRESS (Figure 2). Enriched *Enterobacteriaceae* has been demonstrated to induce inflammatory and stress response [36]. Some studies reported the harmful effect of *Enterobacteriaceae* on cognitive function [37,38], but the role of *Escherichia-Shigella* is unclear. Of note, only 55% percent of OTU2 was *Escherichia-Shigella*; the other 45% is unknown (Supplementary Table S4). Our study also found a negative association between enriched abundance of *Incertae_Sedis* (OTU4) and *Veillonella* (OTU5) and lower abstinence/stress level (NSTRESS) after 14 days, which may indicate that *Incertae_Sedis* and *Veillonella* have a neuro-protective effect. These potential protective effects were supported by previous studies which reported the roles that *Incertae_Sedis* plays in allergic disease [23] and *Veillonella* plays in energy conservation in infants [39]. The roles of *Incertae_Sedis* and *Veillonella* in the first two weeks of NICU hospitalization require further investigation.

Our study found that enrichment of *Enterococcus* (OTU6), the genus level of *Enterococcaceae*, was associated with better handling response (Figure 3). The protective role of *Enterococcaceae* in the gut in cancer patients receiving radiotherapy was reported in a previous study to be involved in maintaining hematopoiesis and intestinal barriers [40]. Elevated abundance of *Enterococcaceae* has been reported to involved in the pathophysiology progression of several disorders such as infection and cytokines response [41,42]. The negative effect of *Clostridium_sensu_stricto_1* (OTU8) on NHANDLING was also confirmed in our study, as it was associated with a higher risk of necrotizing enterocolitis and prematurity [43].

Enriched *Streptococcus* (OTU28) was related to a lower NHANDLING score, indicating better developmental outcomes, and the negative impact accumulated over time in NICU. Aatsinki et al. reported a positive association between behavioral development and *Streptococcus* in infants at the age of 6 months [8], but the role of enriched *Streptococcus* in preterm infants is still unknown. Elevated *Streptococcus* (OTU28) was found in the gut microbiota in children in atopic dermatitis, an allergic reaction [44]. A previous study also reported the role of *Streptococcus* (OTU28) in infections, i.e., sepsis and meningitis in preterm infants [45] and *Streptococcus* pneumoniae in infants [46,47]; a possible reason for this might be related to the immature immune responses [33].

Even the negative effect of *Clostridium_sensu_stricto* (OTU8) and *Streptococcus* (OTU28) on the handling response was identified (Figure 3); our study did not find a significant direct association between breastfeeding and neurobehavioral development. A higher portion of breastfeeding could alter gut microbiota composition [48], in addition to its association with better neurobehavioral outcomes [4]. However, previous studies reported inconsistent findings regarding the effect of breastfeeding on *Clostridium_sensu_stricto* and *Streptococcus*. One study reported that a higher proportion of breastfeeding and human donor milk could significantly increase the enrichment of *Clostridium_sensu_stricto* in preterm infants [49]. Another study also reported the protection role of breastfeeding on decreasing the risk of *Streptococcus* induced infection [50]. Further studies are needed to uncover the entangling between breastfeeding, gut microbiota, and neurobehavior development in preterm infants.

The sex and race differences of neurobehavioral development in preterm infants warrant more effort to investigate the possible underlying mechanisms, even there were no significant findings in the current study. Evidence has confirmed that there exists an impact of sex-dependent gut microbiota on the behavioral development of full-term infants [19,51,52]. The gut microbiota compositions and predicted functional differences between females and males could be a possible reason [18,19,53]. Previous studies also reported on the race differences in gut microbiota diversity [48,54] and composition [54,55]. Future studies should continue to investigate the mechanisms of sex and race disparities of neurobehavioral outcomes in preterm infants.

Our findings provide new evidence to demonstrate the gradually mature brain–gut––microbiome axis contributing to the neurobehavioral development in preterm infants. Manipulating the identified gut microbiota by interventional strategies such as fecal microbiome transplantation and/or supplementing prebiotics and probiotics may effectively improve the measured neurobehavioral outcomes in preterm infants, i.e., stress, handling responses, and quality of movement [56,57,58,59]. The OTUs associated with neurobehavioral development in preterm infants identified in the current study were generated using the 16S RNA sequencing data that may have limitations in conducting data analysis and making inferences based on OTUs, i.e., it is less powerful for detection of differential effects and functions of gut microbiota. Further studies may also need to employ shotgun sequencing and brain imaging techniques to yield more information including the metabolic functions of the gut microbiome community and the activities of the brain–gut–microbiome axis to explore how the gut microbiome and host brain–gut axis function in the growth and development in preterm infants.

To the best of our knowledge, this is one of the first longitudinal studies modeling the impact of early life pain/stress experience and gut microbiota on neurobehavioral outcomes in preterm infants throughout NICU hospitalization. The neurobehavioral development measured by NNNS in this study may serve as valid indicators to predict neurodevelopmental and infant health outcomes in the clinical settings, although it may not directly predict infant mortality or morbidity. One of the limitations of our study was that this study only included preterm infants born at 28 to 32 weeks of gestational age and did not consider extremely preterm infants, who are more likely to have developmental deficits. Another limitation was the weakness of the 16S sequencing data and analysis pipeline based on OTUs. Therefore, generalization of the findings from this study should be cautious, and application of evidence generated in this study should be prudent.

## 5. Conclusions

This study investigated the impact of early life experiences and gut microbiota on neurobehavioral development in preterm infants. The results suggest that cumulative acute painful and stressful experiences may negatively impact neurobehavioral development outcomes. Additionally, certain genera of gut microbiota were found to influence neurobehavioral development, including *Enterobacteriaceae_unclassified*, *Escherichia-Shigella*, *Incertae_Sedis*, *Veillonella*, *Enterococcus*, *Clostridium_sensu_stricto_1*, and *Streptococcus*. These findings suggest that interventions targeting these factors may improve developmental outcomes for preterm infants during their NICU hospitalization. Longitudinal cohort studies such as this one provide valuable insights into the complex interplay between early life experiences and gut microbiota on neurobehavioral development in preterm infants and can inform the development of effective interventions for supporting preterm infant development.

## Figures and Tables

**Table 1:** Characteristics of the included infants, Mean (SD).

	Total (*n* = 55)	Female (*n* = 30)	Male (*n* = 25)
Birth gestational age (weeks)	30.72 (1.71)	30.53 (1.72)	30.96 (1.71)
Birth body length (cm)	39.94 (3.25)	39.56 (3.34)	40.38 (3.17)
Birth body weight (g)	1444.53 (406.61)	1362.87 (413.29)	1542.52 (383.76)
Birth head circumference (cm)	27.86 (1.88)	27.50 (2.05)	28.31 (1.59)
SNAPE II [media, IQR] ^a^	9.31 (9.66)	11.07 (10.57)	7.2 (8.15)
C-section (*n*, %)	34 (61.82%)	21 (70.00%)	13 (52.00%)
Premature rupture of membranes (*n*, %)	22 (40%)	11 (36.67%)	11 (44%)
Race (*n*, %)			
White	41 (74.54%)	23 (76.67%)	18 (72.00%)
American African	11 (20.00%)	4 (13.33%)	7 (28.00%)
Asian	2 (3.64%)	2 (6.67%)	0 (0.00%)
Unknown	1 (1.82%)	1 (3.33%)	0 (0.00%)
Averaged MBM percentage ^b^	61.46% (32.07)	63.09% (33.02)	59.51% (31.46)
Averaged acute pain ^c^	62.66 (9.94)	62.01 (9.65)	63.43 (10.42)
Averaged chronic pain ^c^	89.84 (36.72)	93.44 (37.76)	85.82 (35.68)

^a^ SNAPE II, Score for Neonatal Acute Physiology—Perinatal Extension-II (SNAPPE-II). ^b^ MBM, mother’s breast milk. ^c^ Weighted frequencies (acute) and hours of procedures (chronic) were calculated by timing the counts and intensities of each procedure in each day of NICU stay to generate daily acute pain/stress scores and chronic pain/stress scores.

**Table 2:** Neurobehavioral outcomes of the included infants, Mean (SD).

NNNS ^a^	Total (*n* = 55)	Female (*n* = 30)	Male (*n* = 25)
Stress/abstinence (NSTRESS)	0.18 (0.09)	0.19 (0.09)	0.17 (0.09)
Handling (NHANDLING)	0.56 (0.21)	0.57 (0.21)	0.56 (0.22)
Quality of movement (NQMOVE)	3.97 (0.62)	3.90 (0.65)	4.05 (0.60)

^a^ NNNS, NICU Network Neurobehavioral Scale.

## Data Availability

The raw sequence data were archived in NCBI (https://submit.ncbi.nlm.nih.gov/subs/sra/SUB8904718/). Deidentified data are available upon reasonable request. Requests to access these datasets should be directed to xiaomei.cong@yale.edu.

## Supplementary Materials

The following supporting information can be downloaded at: https://www.mdpi.com/article/10.3390/microorganisms11030814/s1. Figure S1: CONSORT form; Figure S2: Feeding and NISS; Figure S3: Daily feeding patterns for females and males; Figure S4: Neurobehavioral development; Table S1: Daily sample collection of each infant; Table S2: Taxonomy of each OTU; Table S3: Estimation of Control Variables: Results of regression models; Table S4: Selected 8 OTUs associated with of NNNS subscales; Supplementary File S1: Coding system for events of different levels of pain/stress in NISS assessment; Supplementary File S2: Raw sequence data processing.
