# Supplementary material for: The Impact of Early Life Experiences and Gut Microbiota on Neurobehavioral Development in Preterm Infants: A Longitudinal Cohort Study
Source: Microorganisms. 2023 Mar 22;11(3):814. doi: 10.3390/microorganisms11030814 (PMC10056840; doi:10.3390/microorganisms11030814)
Supplement: Supplementary file 1 [file microorganisms-11-00814-s001.zip › Table S3 Estimation of Control Variables.pdf]

Table S3 Estimation of Control Variables

	GA	Female vs. Male	White vs. Non-White	C-section vs. Vaginal	PROM vs. N-PROM	MBM proportion	Acute pain/stress	Chronic pain/stress	kangaroo care	Antibiotics use in the first 3 days	Intercept
NSTRESS	-0.012	0.019	-0.007	0.018	-0.007	-0.061	0.002	0.001	-0.001	0.048	0.495
NHANDLING	-0.043	0.007	-0.041	0.103	-0.05	0.001	0.008	-0.029	-0.001	0.088	1.294
NQMOVE	0.128	-0.112	0.389	-0.199	0.148	0.268	-0.004	0.024	0.007	-0.314	-0.455

Note: PROM, Pre-rupture of membrane; MBM, Mother’s Breast Milk.
