# Supplementary material for: The Impact of Early Life Experiences and Gut Microbiota on Neurobehavioral Development in Preterm Infants: A Longitudinal Cohort Study
Source: Microorganisms. 2023 Mar 22;11(3):814. doi: 10.3390/microorganisms11030814 (PMC10056840; doi:10.3390/microorganisms11030814)
Supplement: Supplementary file 1 [file microorganisms-11-00814-s001.zip › Table S4 Selected 8 OTUs associated with of NNNS subscales.pdf]

Table S4 Selected 8 OTUs associated with of NNNS subscales

NSTRESS	NHANDLING	NQMOVE	OTU	Size	Taxonomy
Selected	Selected	NA	Otu00001	13859029	Bacteria(100);Proteobacteria(100);Gammaproteobacteria(100);Enterobacteriales(100);Enterobacteriaceae(100);Enterobacteriaceae_unclassified(95);
Selected	Selected	NA	Otu00002	9732098	Bacteria(100);Proteobacteria(100);Gammaproteobacteria(100);Enterobacteriales(100);Enterobacteriaceae(100);Escherichia-Shigella(55);
Selected	NA	Selected	Otu00004	2880234	Bacteria(100);Firmicutes(100);Clostridia(100);Clostridiales(100);Peptostreptococcaceae(100);Incertae_Sedis(100);
Selected	Selected	NA	Otu00005	2667454	Bacteria(100);Firmicutes(100);Negativicutes(100);Selenomonadales(100);Veillonellaceae(100);Veillonella(100);
NA	Selected	NA	Otu00006	2390001	Bacteria(100);Firmicutes(100);Bacilli(100);Lactobacillales(100);Enterococcaceae(99);Enterococcus(99);
Selected	Selected	NA	Otu00008	1576632	Bacteria(100);Firmicutes(100);Clostridia(100);Clostridiales(100);Clostridiaceae_1(100);Clostridium_sensu_stricto_1(100);
Selected	NA	NA	Otu00017	168136	Bacteria(100);Proteobacteria(100);Gammaproteobacteria(100);Enterobacteriales(100);Enterobacteriaceae(100);Enterobacteriaceae_unclassified(100);
NA	Selected	NA	Otu00028	92536	Bacteria(100);Firmicutes(100);Bacilli(100);Lactobacillales(100);Streptococcaceae(100);Streptococcus(100);

Note: NA, not applicable.
