# Supplementary figures and images for: The Impact of Early Life Experiences and Gut Microbiota on Neurobehavioral Development in Preterm Infants: A Longitudinal Cohort Study
Source: Microorganisms. 2023 Mar 22;11(3):814. doi: 10.3390/microorganisms11030814 (PMC10056840; doi:10.3390/microorganisms11030814)

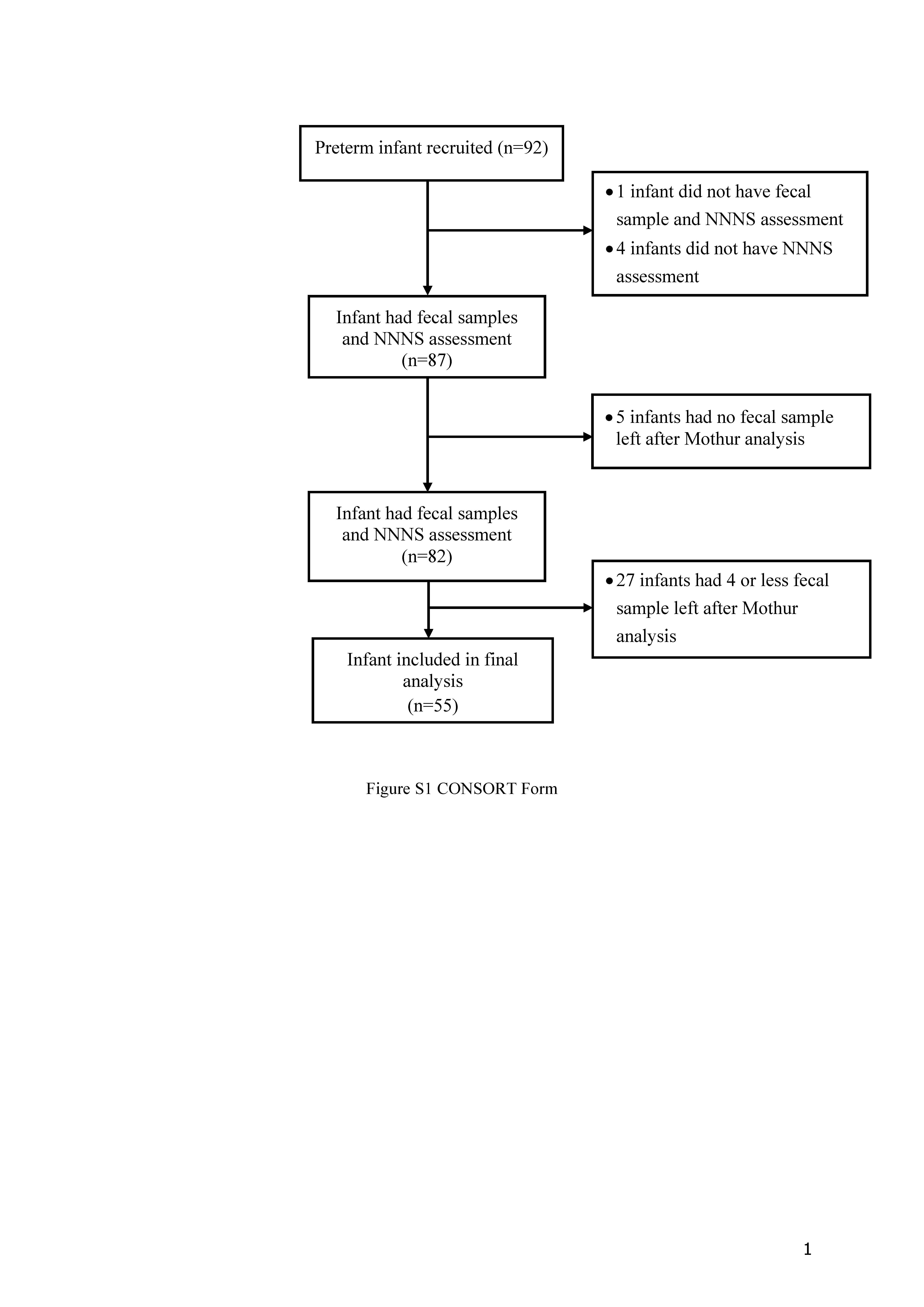

Supplement: Supplementary file 1 [file microorganisms-11-00814-s001.zip › Figure S1 CONSORT Form.tiff]

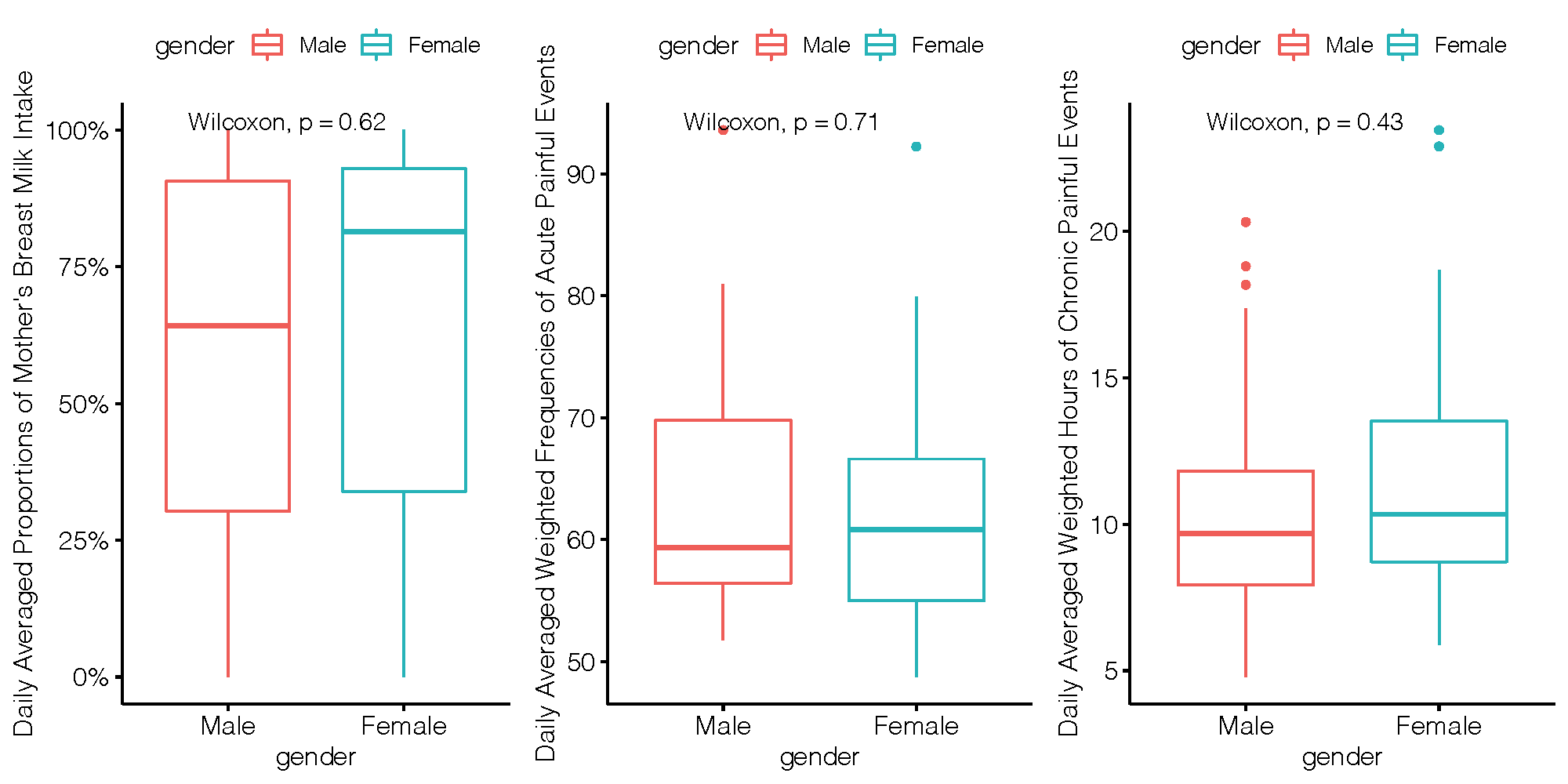

Supplement: Supplementary file 1 [file microorganisms-11-00814-s001.zip › Figure S2.tiff]

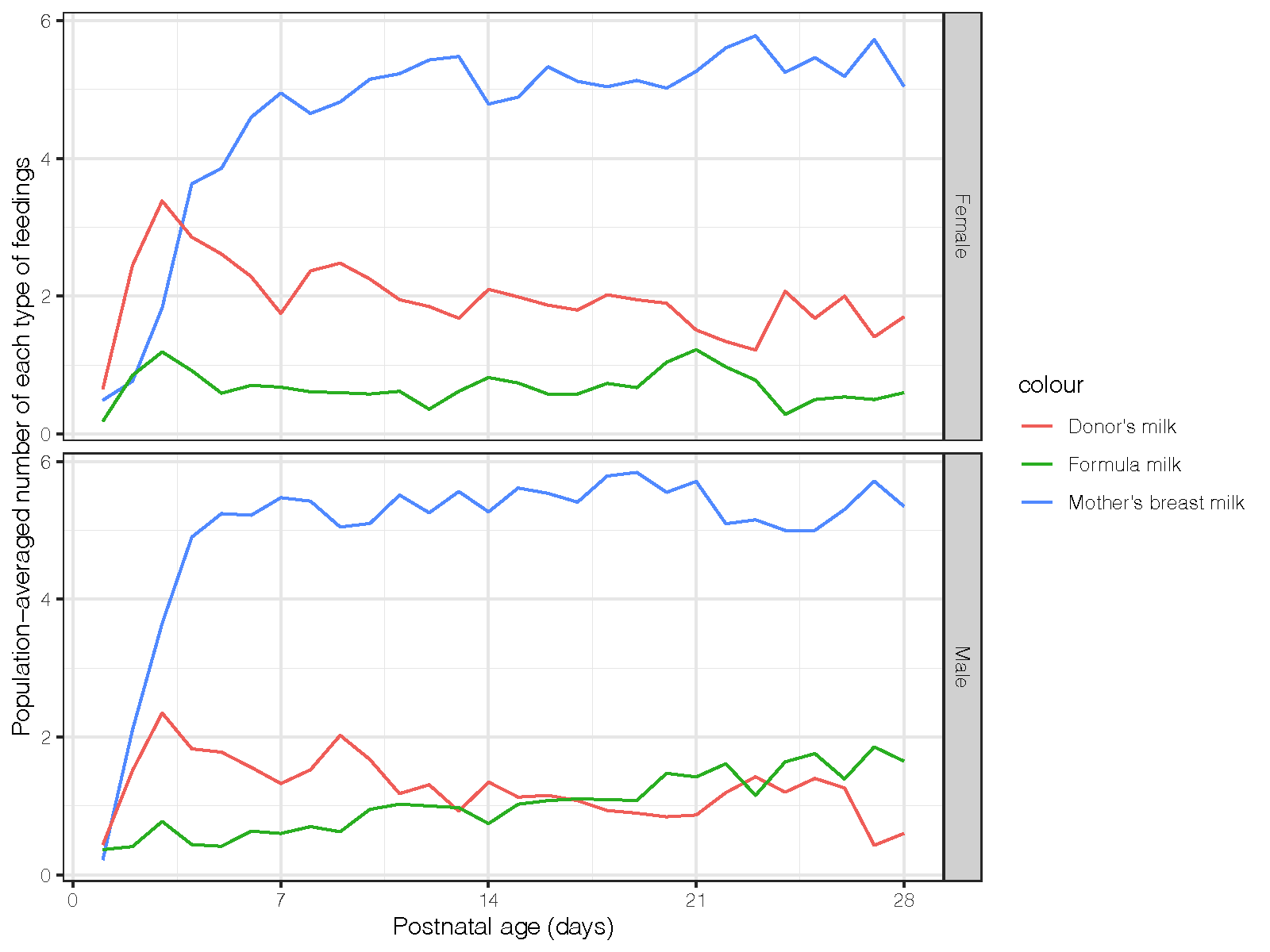

Supplement: Supplementary file 1 [file microorganisms-11-00814-s001.zip › Figure S3 feed.pna.tiff]

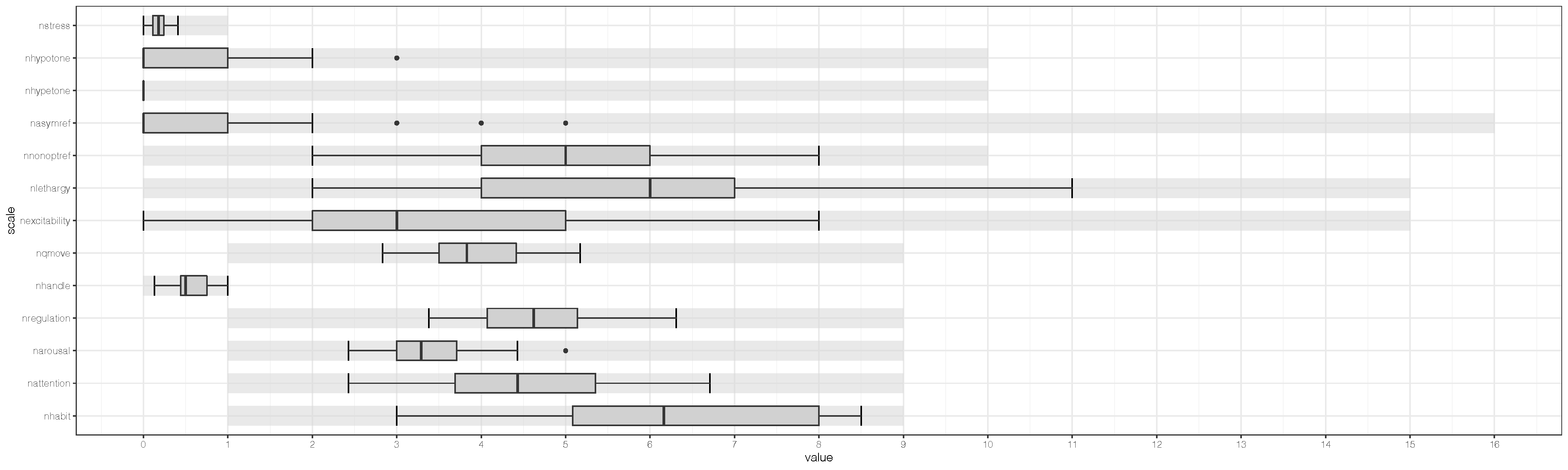

Supplement: Supplementary file 1 [file microorganisms-11-00814-s001.zip › Figure S4 nnns.tiff]
